# Maternal Hepatitis B Virus or Hepatitis C Virus Carrier Status and Long-Term Endocrine Morbidity of the Offspring—A Population-Based Cohort Study

**DOI:** 10.3390/jcm9030796

**Published:** 2020-03-14

**Authors:** Naim Abu Freha, Tamar Wainstock, Tzvi Najman Menachem, Eyal Sheiner

**Affiliations:** 1 The Institute of Gastroenterology and Hepatology, Soroka University Medical Center and the Faculty of Health Sciences, Ben-Gurion University of the Negev, Beer-Sheva 84101, Israel; 2 The Department of Public Health, Faculty of Health Sciences, Ben-Gurion University of the Negev, Beer-Sheva 84101, Israel; wainstoc@bgu.ac.il; 3 Medical School for International Health, Ben-Gurion University of the Negev, Beer-Sheva 84101, Israel; najman@post.bgu.ac.il; 4 Department of Obstetrics and Gynecology, Soroka University Medical Center, Ben-Gurion University of the Negev, Beer-Sheva 84101, Israel; sheiner@bgu.ac.il

**Keywords:** Hepatitis B, Hepatitis C, endocrine morbidity, long term, offspring

## Abstract

This study aimed to investigate the long-term effect of maternal hepatitis B virus (HBV) or hepatitis C virus (HCV) carrier status on offspring endocrine morbidity. A population-based cohort study included all singleton deliveries between the years 1991–2014 at the Soroka University Medical Center, Beer-Sheva, Southern Israel. The mothers were subdivided into three groups, HBV carriers, HCV carriers and non-carriers. Data regarding the long-term endocrine morbidity of their offspring were compared between the groups. The study included 242,905 (99.7%) non-carrying mothers, 591 (0.2%) mothers who were carriers for HBV and 186 (0.1%) mothers who were carriers for HCV. The Kaplan–Meier’s survival curve demonstrated a significantly higher cumulative endocrine morbidity in children born to mothers with HCV (log-rank test, *p* = 0.002). Specifically, higher rates of hypoglycemia were noted among the offspring born to mothers who were carriers of HCV (1.1%; *p* = 0.001) compared with the offspring of mothers who were either carriers of HBV (0.2%) or non-carriers (0.1%). A Cox regression model controlled for maternal age, gestational age, maternal diabetes, hypertensive disorders of pregnancy, found maternal HCV carrier status to be independently associated with pediatric endocrine morbidity in the offspring (adjusted hazard ratio = 5.05, 95% CI: 1.625–15.695, *p* = 0.005). Maternal HCV carrier status is an independent risk factor for long-term endocrine morbidity.

## 1. Introduction

Hepatitis B virus (HBV) and hepatitis C (HCV) infection are relatively common chronic liver infections among young people, including women of childbearing age. The relationship between pregnant women, their fetuses, and the hepatitis virus is a complex process of delicate interactions [1,2]. A previous study found a prevalence of 0.4% carrier status for hepatitis B or C among pregnant women [3]. Furthermore, the carrier status of chronic HBV or HCV was found to be an independent risk factor for adverse perinatal and maternal outcomes [3].

Chronic viral hepatitis, particularly HCV, is known to be associated with variable extrahepatic manifestations such as endocrine dysfunction, an increased risk of thyroid disease, diabetes mellitus and hepatic steatosis [4,5,6,7,8,9,10]. Additionally, infection with HCV is frequently associated with high titers of anti-thyroid antibodies that further increase the patient’s risk for developing hypothyroidism [11,12].

The liver plays a key role in fuel metabolism and is a major site of protein synthesis and hormone regulation. As the key metabolic regulator of the body, the liver also plays a critical role in the development of endocrine disorders such as diabetes mellitus and obesity [13].

A long-standing HCV infection with an associated chronic necroinflammation of the liver increases the risk of developing specific endocrine diseases [11,12]. An important uninvestigated issue regarding chronic HBV or HCV during pregnancy is the long-term effect on the endocrine morbidity of the offspring. This study was conducted to investigate the association between maternal HBV or HCV carrier status and long-term endocrine morbidity among their offspring.

## 2. Materials and Methods

### 2.1. Setting

A population-based cohort analysis was performed with the data on deliveries in the Soroka University Medical Center (SUMC). SUMC is a tertiary, 1100-bed hospital located in the city of Beer-Sheva, Israel, that houses the largest birth center in the country. Additionally, the SUMC is the only hospital with an obstetrics ward and a pediatric ward in the Negev region (southern Israel). The study was approved by the local institutional review board (IRB) committee.

### 2.2. Study Population

In this retrospective study, the cohort included all singleton infants born between the years 1991 and 2014 who were discharged alive from the SUMC and who were born to HBV- or HCV-carrying mothers (HBsAg and/or anti-HCV seropositive) according to the diagnosis list during the pregnancy or delivery hospitalization. This cohort was compared with non-exposed children (delivered to non-carriers).

### 2.3. Data Collection

Data were collected from two databases that were cross-linked and merged (based on patient national identification number), the computerized pediatric hospitalization database of the SUMC (“Demog-ICD9”) and the computerized perinatal database of the obstetrics and gynecology department. The computerized perinatal database consists of information recorded directly after delivery by an obstetrician. Clinical and demographic data relating to the mother, including age, parity, gestational and pregestational diabetes mellitus, hypertensive disorders (chronic or gestational hypertension and preeclampsia with or without severe features), gestational age and gender, were compiled. In addition, we collected data regarding the gravidity group, which was defined as the number of pregnancies, including miscarriages. Clinical data pertaining to the newborns, including Apgar score 1 and Apgar score 5, birth weight, low birth weight (LBW) defined as under 2500 g, and perinatal mortality, were also collected. Multiple pregnancies and fetuses with congenital malformations were excluded from the study. Long-term data regarding endocrine morbidity during hospitalizations for endocrine, or any other cause, of the children up to the age of 18 were collected according to a set of ICD-9 codes detailed in the Supplementary Table (Table S1). The carrier status of hepatitis was considered to be the independent variable, while the endocrine morbidity during the hospitalizations of the children was considered as the outcome variable (the dependent variable). Patient follow-up was terminated after the child’s first hospitalization for any endocrine morbidity, after the child reached 18 years of age or after any hospitalization resulting in the child’s death.

### 2.4. Statistical Analysis

Patient characteristics were presented as the mean ± SD for continuous variables and as percentages for the categorical variables. The chi-square test was used to test the association between categorical variables (the exposure and background variables). In variables with <5 observations per cell, the Fischer exact chi-square test was used. A Kaplan–Meier survival curve was used to compare cumulative hospitalization incidence over the time period. Only the first admission with any endocrine-related condition for a given individual was included in the survival analysis. In our study, we followed the offspring until their first hospitalization, at which point we excluded them from the follow-up (since it was already captured as morbidity). This is due to the fact that the same child could be hospitalized multiple times. Children without hospitalizations remained in follow-up until the age of 18. The differences between the curves were assessed using the log-rank test. To investigate the association between maternal HBV or HCV carrier status and the long-term endocrine morbidity among their offspring, a Cox proportional hazards regression model was estimated. The regression model was adjusted for confounding and clinically significant variables, such as maternal age, maternal hypertensive disorders of pregnancy (chronic or gestational hypertension and preeclampsia with or without severe features) and maternal diabetes (pre-gestational and gestational). Deliveries by non-exposed mothers were used as a point of reference. All analyses used two-sided tests and a 5% significance level. A statistical analysis was performed using the SPSS package 23rd edition (IBM/SPSS, Chicago, IL, USA).

## 3. Results

In the study period, 243,682 deliveries occurred. Of those, there were 591 (0.2%) and 186 (0.1%) deliveries by mothers who were carriers of hepatitis B and hepatitis C, respectively.

Demographic details such as maternal and perinatal characteristics organized by hepatitis status (HBV, HCV, non-carrier) are summarized in Table 1. Significant differences between carriers and non-carriers were found regarding maternal age at birth, gestational age, hypertensive disorders, preterm delivery and birth weight.

The long-term endocrine morbidities and hospitalizations during the follow-up period (0–18 years, median 10.22) in offspring born to chronic hepatitis carrying mothers compared to offspring born to non-hepatitis carriers are summarized in Table 2. We found a significantly higher rate of hospitalization due to hypoglycemia in the offspring of HCV carriers compared to the offspring of HBV carriers and non-carriers, 1.1% vs. 0.2% and 0.1%, *p* = 0.001, respectively. No other significant differences (such as thyroid disease, obesity, adrenal diseases, diabetes mellitus and all-cause endocrine hospitalization) were found among hepatitis carriers compared with the non-carriers.

**Figure 1:**
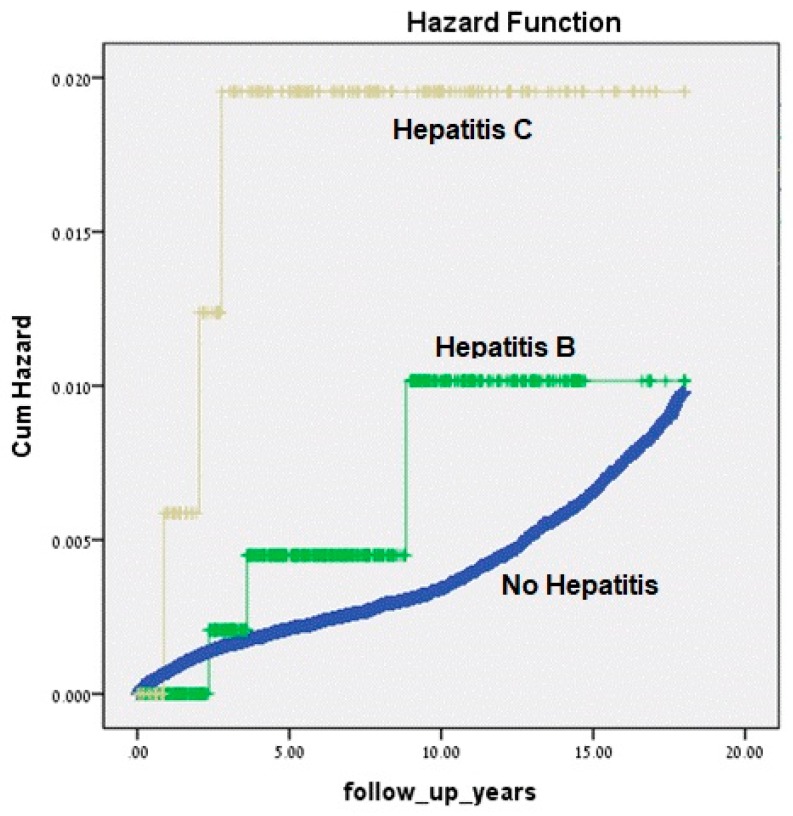
Kaplan–Meier survival curve demonstrating the cumulative incidence of hospitalizations involving endocrine morbidity in children born to mothers carrying chronic hepatitis C, B or no hepatitis during the pregnancy.

In the Kaplan–Meier survival curve (Figure 1), the children born to HCV-carrier mothers showed a significantly higher cumulative incidence of endocrine-related hospitalizations when compared with children born to non-carrying mothers (Kaplan–Meier, log-rank *p* = 0.002).

The Cox regression model estimation results, shown in Table 3, present the association between maternal chronic hepatitis during pregnancy and the long-term endocrine-related hospitalizations in children up to 18 years old. As shown in the model, chronic maternal HCV infection during pregnancy was found to be a significant and independent risk factor for the long-term endocrine-related hospitalization of the offspring, with an adjusted hazard ratio (HR) of 5.05 (95% CI: 1.625–15.695, *p* < 0.005).

## 4. Discussion

The most important finding of the present population-based study is that the maternal HCV carrier status is an independent risk factor for the long-term endocrine morbidity of the offspring. The results remained significant after controlling for critical confounders, such as diabetes mellitus [14], a well established risk factor of long-term endocrine morbidity in the offspring as well as other confounders that might contribute to this association such as gestational age [15], birth weight or chronic hepatitis among the children. To the best of our knowledge, there are no other reports investigating long-term endocrine morbidity among offspring of chronic hepatitis B or C carrier status.

When comparing HBV and HCV, there are several similarities. On the one hand HBV and HCV are similar with regard to their target cells and their disease progression. However, the viruses differ in terms of the symptoms they cause, the immune response they precipitate, and the tactics they use to persist in host cells. For example, HCV infections are typically subclinical and result in vigorous innate immune activation, while HBV infections are typically more acute, but less likely to cause chronic hepatic infection if contracted in adult life. Additionally, the symptoms of HBV (when compared to HCV) engage the innate immune system and associated inflammatory response to a lesser extent [16].

Furthermore, HCV and HBV differ in their extrahepatic manifestation in carriers. HCV is known to be associated with thyroiditis, hypothyroidism, lymphoma, cryoglobulinemia, non-alcoholic fatty liver, diabetes mellitus, gondadal dysfunction and obesity [17].

In addition to the potential synergistic effects that HCV and metabolic syndrome have on increasing liver complications, the interaction between HCV, glucose and lipid metabolism must also be considered [18]. The latter interaction has been shown to cause hepatitis with extrahepatic insulin resistance. It can also accelerate complications of liver disease and its extrahepatic complications, for example, increasing the risk of cardiovascular disease [18].

These differences may explain the relationship between endocrine morbidity among offspring born to HCV carrying mothers and the lack of this relationship among the offspring born to HBV-carrying mothers.

To fully understand the interaction between chronic HCV infection and endocrine morbidity among the offspring, it is imperative to understand how the immune system and the inflammatory response, combined with poor perinatal outcomes, relate to the underlying maternal HCV infection.

Autoimmunity in HCV-carrying mother increases the risk of anti-thyroid antibodies, which may get passed on to the offspring and put the child at an increased risk of developing hypothyroidism [11,12].

Additionally, the immune response to chronic infections of hepatitis C is associated with increased levels of interferons and pro-inflammatory cytokines resulting from the initiation of signal transduction pathways [19]. Some of these proteins may be able to cross the placenta and could contribute to the relationship modeled by this study.

Lastly, chronic HCV infection by itself is associated with elevated risk of poor perinatal outcomes, as was previously demonstrated [3]. These poor outcomes, such as low birth weight, have been found to be associated with adverse endocrine morbidity among offspring [20].

The combination of the above-mentioned aspects, combined with the effects of chronic HCV carrier status in mothers and the in-utero exposure of the infants to those associated immune agents, have an important role in the long-term endocrine health of the offspring and may explain their increased morbidity.

We recently published two other works that show that the hepatitis B/C carrier status of the mothers is an independent risk factor for long-term gastrointestinal and respiratory morbidity among the offspring [21,22]. In the present study, we found that maternal chronic hepatitis C, which is a known risk factor of endocrine morbidity to the carrier, can affect long-term endocrine morbidity among their offspring. Thus, we believe that primary prevention and screening is important, particularly among high-risk groups and pregnant women. Universal screening of pregnant women for hepatitis is recommended by different societies [23,24]. Screening women of reproductive age for hepatitis before considering pregnancy might be helpful. If diagnosed, this would encourage infected women to seek out highly effective, short length (8–12 weeks) direct-acting antiviral (DAAS) treatment before conception. The usefulness of such screening lies not only in the personal risk reduction of hepatitis C complications (cirrhosis, hepatocellular carcinoma, etc.) but also in the decreased risk of vertical transmission and the long-term morbidity of their offspring. The strength of our study lies in the large number of enrolled subjects and the long-term follow-up of the offspring. The limitations of our study lie firstly in the retrospective design of the study, and secondly in the lack of accurate data regarding certain antibody titers (such as anti-thyroid antibodies) among the mothers and children. Another limitation is the lack of accurate data on treatments, viral load and fibrosis grade among hepatitis carriers.

To conclude, maternal HCV carrier status was found to be an independent risk factor for the long-term endocrine morbidity of offspring after controlling for confounders. To the best of our knowledge, this is the first study that investigated long-term endocrine morbidity among the offspring born to mothers carrying hepatitis to have found this association. Additional studies are needed to further investigate this association to better our understanding of the pathophysiology of this association before practical guidelines for the long-term follow-up of the offspring born to HCV carriers can be established.

## Figures and Tables

**Table 1:** Demographics, maternal and perinatal characteristics by study group.

Characteristics	No Hepatitis *n* (%), 242,905 (99.7)	Hepatitis B *n* (%), 591 (0.2)	Hepatitis C *n* (%), 186 (0.1)	*p*-Value
Mother’s age at birth (years ± SD)	28.16 ± 5.8	28.8 ± 5.9	30.6 ± 5.1	<0.001 *
Gestational age-Weeks	39.1 ± 1.9	38.8 ± 2	38.2 ± 2.4	<0.001 *
Gravidity group				
1	47,872 (19.7)	120 (20.3)	39 (21)	0.972 **
2–4	116,182 (47.8)	282 (47.7)	85 (45.7)	
5+	78,808 (32.4)	189 (32)	62 (33.3)	
Diabetes Mellitus (gestational and pre-gestational)	12,120 (5)	30 (5.1)	9 (4.8)	0.991 **
Hypertensive disorders of pregnancy	12,191 (5)	41 (6.9)	15 (8.1)	0.017 **
Preterm delivery				
<37	16,647 (6.9)	46 (7.8)	27 (14.5)	<0.001 **
<34	3289 (1.4)	12 (2)	8 (4.3)	0.001 **
Birth weight				
Low ≤ 2500 g	16,330 (6.7)	42 (7.1)	32 (17.2)	<0.001 **
very low ≤ 1500 g	1454 (0.6)	6 (1)	2 (1.1)	0.298 ***
Infant Gender				
Male	123,468 (50.8)	320 (54.1)	89 (47.8)	0.196 **
Apgar 1 min < 7	12,947 (5.3)	33 (5.6)	10 (5.4)	0.963 **
Apgar 5 min < 7	5,497 (2.3)	9 (1.5)	3 (1.6)	0.403 ***
Birth-weight	3205 ± 528	3236 ± 528	3065 ± 634	<0.001 *
Perinatal mortality	1,334 (0.5)	3 (0.5)	3 (1.6)	0.145 ***

* results of an ANOVA test, ** results of a Pearson’s chi-square test, *** results of a Fischer chi-square test.

**Table 2:** Long-term endocrine morbidities and hospitalization in children (up to the age of 18 years) born to chronic hepatitis-carrier mothers compared with non-carriers.

Endocrine Morbidity	No hepatitis *n* = 241,571 (%)	Hepatitis B *n* = 588 (%)	Hepatitis C *n* = 183 (%)	*p*-Value
Thyroid Disease	110 (0.04)	0 (0)	0 (0)	0.839
Diabetes mellitus	215 (0.09)	0 (0)	1 (0.55)	0.090
Hypoglycemia	287 (0.12)	1 (0.17)	2 (1.1)	0.001
Obesity	435 (0.18)	2 (0.34)	0 (0)	0.558
Adrenal disease	43 (0.018)	0 (0)	0 (0)	0.934
Endocrine hospitalization	1145 (0.47)	3 (0.51)	3 (1.64)	0.072

**Table 3:** Cox regression model estimation results: adjusted hazard ratios and respective 95% confidence interval

Variables	Adjusted HR	95% CI	*p*-Value
Hepatitis B	1.8	0.607–5.859	0.273
Hepatitis C	5.05	1.625–15.695	0.005
Maternal age at birth (years)	1.0	0.992–1.012	0.653
Diabetes mellitus (gestational and pregestational)	1.82	1.494–2.219	<0.001
Hypertensive disorders of pregnancy	1.308	1.053–1.625	0.015
Gestational age (weeks)	0.935	0.913–0.957	<0.001

## Supplementary Materials

The following are available online at https://www.mdpi.com/2077-0383/9/3/796/s1, Table S1: ICD-9 codes of endocrine disease.
