# Supplementary material for: Maternal Hepatitis B Virus or Hepatitis C Virus Carrier Status and Long-Term Endocrine Morbidity of the Offspring—A Population-Based Cohort Study
Source: J Clin Med. 2020 Mar 14;9(3):796. doi: 10.3390/jcm9030796 (PMC7141343; doi:10.3390/jcm9030796)
Supplement: Supplementary file 1 [file jcm-09-00796-s001.pdf]

Journal of
Clinical Medicine

Supplementary Table S1 ICD-9 codes of endocrine diseases

	Code	Diagnosis description
Hypothyroidism	243	Congenital hypothyroidism
	2449	Unspecified acquired hypothyroidism
Diabetes mellitus—type I	25001	Juvenile type diabetes mellitus without mention of complication
	25001	Type I (juvenile type), diabetes mellitus without complication, not stated as uncontrolled
	25003	Diabetes mellitus without complication, type I, uncontrolled
	25011	Juvenile type diabetes mellitus with ketoacidosis
	25011	Type I (juvenile type) ,diabetes mellitus with ketoacidosis, not stated as uncontrolled
	25013	Diabetes with ketoacidosis type I, uncontrolled
	25031	Juvenile diabetes, not stated as uncontrolled, with other coma
	25041	Type I (juvenile type) diabetes with renal manifestations not stated as uncontrolled
	25081	Juvenile diabetes, not stated as uncontrolled, + specified manifestation
	25081	Type I (juvenile type), diabetes with specified manifestation not stated as uncontrolled
	25083	Juvenile diabetes, uncontrolled, + specified manifestations
Diabetes mellitus—type II or NOS	2500	Diabetes mellitus without mention of complication
	2508	Diabetes with other specified manifestations
	24900	Secondary diabetes mellitus without mention of complication, not stated as uncontrolled, or unspecified
	24901	Secondary diabetes mellitus without mention of complication uncontrolled
	25000	Type II/unspecified type, diabetes mellitus without complication, not stated as uncontrolled
	25002	Type II or unspecified type, diabetes mellitus, uncontrolled
	25010	Type II or unspecified type, diabetes mellitus with ketoacidosis, not stated as uncontrolled
	25050	Adult-onset diabetes, not stated as uncontrolled, + ophthalmic manifestation
	25050	Type II or unspecified type, diabetes with ophthalmic manifestation not stated as uncontrolled
	25080	Type II or unspecified type, diabetes with specified manifestation not stated as uncontrolled
Hypoglycemia	2510	Hypoglycemic coma (nondiabetes insulin coma)
	2511	Other specified hypoglycemia
	2512	Hypoglycemia unspecified
Hyperlipidemia	2721	Pure hyperglyceridemia
	2722	Mixed hyperlipidemia
	2724	Other and unspecified hyperlipidemia
Overweight and obesity	2779	Unspecified disorder of metabolism
	2780	Overweight and obesity
	2781	Localized adiposity
	27800	Obesity, unspecified
	27801	Morbid obesity
	27802	Overweight
